# CD147 as a Novel Prognostic Biomarker for Hepatocellular Carcinoma: A Meta-Analysis

**DOI:** 10.1155/2017/5019367

**Published:** 2017-03-12

**Authors:** Fei Peng, Hui Li, Qian You, Hongru Li, Dongwen Wu, Chunxiang Jiang, Guangtong Deng, Yan Li, Yuyan Li, Yi Wu

**Affiliations:** ^1^Department of Laboratory, The First Affiliated Hospital of Hunan Normal University, Hunan Provincial People's Hospital, Changsha, Hunan, China; ^2^Reproductive Department, Xiangya Hospital, Central South University, Changsha, China; ^3^Xiangya School of Medicine, Central South University, Changsha, China; ^4^Hepatic Surgery Department of Xiangya Hospital, Central South University, Changsha, China; ^5^Department of Pediatrics, The First Affiliated Hospital of Hunan Normal University, Hunan Provincial People's Hospital, Changsha, Hunan, China

## Abstract

We conducted a meta-analysis to investigate the controversial association of CD147 expression with HCC prognosis and clinicopathological characteristics. Eight studies from PubMed (1966–2016), EMBASE (1980–2016), Cochrane Library (1996–2016), Web of Science (1945–2016), China National Knowledge Infrastructure (1982–2016), and Wanfang databases (1988–2016) were considered. The associations between CD147 expression and clinicopathological parameters and overall survival (OS) or DFS/RFS were reassessed using the meta-analysis for odds ratio (OR) or hazard ratio (HR) and 95% confidence interval (CI). CD147 expression was associated with DFS/RFS (HR = 3.26; 95% CI: 1.82–5.83; *P* < 0.0001) but not with OS (HR = 1.35; 95% CI: 0.56–3.29; *P* = 0.51). We also delved deeper into the association between median survival time and CD147 expression owing to significant heterogeneity and found significant differences between high and low CD147 expression groups with respect to median survival time. CD147 expression was closely associated with the TNM stage (OR = 0.18; 95% CI: 0.04–0.85; *P* = 0.03) and venous invasion (OR = 6.29; 95% CI: 1.70–23.20; *P* = 0.006). In contrast, there was no association between CD147 expression and tumor stage, cirrhosis, differentiation, lymph node metastasis, HBsAg, and serum AFP levels. Thus, CD147 expression is potentially closely related to HCC survival and associated clinicopathological parameters, paving the way for further research.

## 1. Introduction

Hepatocellular carcinoma (HCC) is the sixth most prevalent malignancy and the third leading cause of cancer-related death worldwide [1]. Although incidence rates have been declining for most cancers, rates are increasing for HCC [2]. In spite of its increased incidence, there is only basic understanding of disease pathogenesis and there are limited therapeutic options [3]. The 5-year overall survival rate of individuals with HCC is only 8.9%, and this has barely improved over the past two decades [4]. Recently, many prognostic markers, such as CD133, CD44, keratin 19, Serum M65, and serum sCD163, have been introduced to help identify patients who are likely to have a poor prognosis and benefit from more aggressive treatment approaches [5–8].

CD147 is also known as HAb18G in humans [9, 10]. As a transmembrane glycoprotein and a member of immunoglobulin superfamily, it was first named as tumor cell-mediated collagen enzyme activation factor (tumor cell collagenase stimulatory factor, TCSF) and later on renamed as EMMPRIN [11]. Earlier studies demonstrated that the CD147 molecule was highly expressed on the surface of various cancer cells, including cancers of the liver, lung, breast, kidney, colon, prostate, and esophagus [12]. There is emerging evidence indicating that CD147 plays a central role in the progression of many cancers due to increased adhesion, migration, invasion, and matrix metalloproteinases [13–16]. Importantly, increased expression of cancer-associated CD147 predicts aggressive behavior and poor prognosis [12, 17–20].

Recent reports have indicated that the expressions of CD147 correlate with poor clinical factors and outcomes in hepatic carcinoma [21]. However, another study has nullified this hypothesis [18]. Therefore, we conducted this meta-analysis for the quantitative inspection of the relationship between CD147 expression and clinicopathological features and survival of hepatic carcinoma patients.

## 2. Materials and Methods

The following were the criteria for the inclusion of studies in our analysis.

(1) The studies had to be published or unpublished case control study or cohort study in English or Chinese with the full text available. (2) All cases had complete clinicopathological characteristic data, without radiotherapy or chemotherapy or biological therapy before sampling. (3) Diagnosis of hepatic carcinoma cancer was proven by pathological methods. (4) Studies must have CD147 expression analyzed by immunohistochemical staining in primary hepatic carcinoma tissue (via either biopsy or surgical) and not in serum or any other kind of specimen. (5) The best quality study was retained for conducting duplicated study.

The following were the criteria for the exclusion of studies in our analysis: (1) cell or animal studies, case reports, letters, and reviews; (2) the standard of pathological diagnosis being not clear.

### 2.1. Search Strategy

The studies were included from PubMed (1966–2016), EMBASE (1980–2016), Cochrane Library (1996–2016), Web of Science (1945–2016), China National Knowledge Infrastructure (1982–2016), and Wanfang databases (1988–2016). The studies were restricted to humans, but not by date, language, or publication status. The following combined search terms were used: (Liver Neoplasms OR hepatic neoplasm^*∗*^ OR hepatocellular cancer^*∗*^ OR hepatic cancer^*∗*^ OR liver cancer^*∗*^) AND (CD147 OR extracellular matrix metalloproteinase inducer OR EMMPRIN) AND (prognosis OR survival OR outcome OR prognostic). We combined the terms appropriately with MeSH Terms and used an appropriate adjustment for different databases. Details of the search strategies can be found in Appendix  1 (see Supplementary Material available online at https://doi.org/10.1155/2017/5019367).

### 2.2. Statistical Analysis

The records were independently scanned by two authors to exclude irrelevant studies. Then, full-text articles were independently excluded, and controversial opinions were resolved by the third author. All of the data were extracted independently by two authors. The Newcastle-Ottawa Scale (NOS) [22] was applied to assess the included studies. RevMan 5.3 software and Stata 13.0 software were used for analysis. For each study, the HR was estimated by a method that was dependent on the results provided in the publication. The most accurate method was to retrieve the HR estimate and its variance from the reported results or to calculate it directly using parameters provided by the authors for univariate analysis. If an article described both univariate and multivariate factors, we chose the latter as the survival in HCC is affected by a combination of factors. Otherwise, Kaplan-Meier curves were read using Engauge Digitizer version 4.1 [23], which can estimate a relatively accurate HR [24, 25], with the assumption that, during the study follow-up, the rate of patients censored was constant. If this method was used, three independent persons read the curves to reduce the variation. Hazard ratios (HR) and 95% confidence intervals (95% CI) were used to evaluate the relationship between CD147 expression among OS (overall survival) and DFS/RFS (disease-free survival/recurrence-free survival). Median survival ratio (MSR) and 95% CI were used to evaluate the median survival time. Moreover, we also examined the correlation between CD147 expression and the clinical variables in liver cancer through odds ratio (OR). Fixed-effects model was adopted for studies without significant heterogeneity (*P* > 0.1 and *I*^2^ < 50%); otherwise, random-effects model was applied. Wherever possible, heterogeneity was explored and subgroup analyses were performed according to follow-up time, the nature of HR (multivariate or univariate), liver transplantation status, and cut-off value. These aspects may influence our conclusion about the association between CD147 and survival of patients with HCC.

Sensitivity analysis was performed to evaluate the influences of individual studies on the final effect size. Egger's test was used to assess publication bias (*P* < 0.05 was considered statistically significant). If publication bias was confirmed and the data were enough, a trim-and-fill method developed by Duval and Tweedie was implemented to adjust for this bias [26]. Then, we replicated the funnel plot with its “missing” counterparts around the adjusted summary estimate.

## 3. Results

### 3.1. Study Characteristics

**Figure 1:**
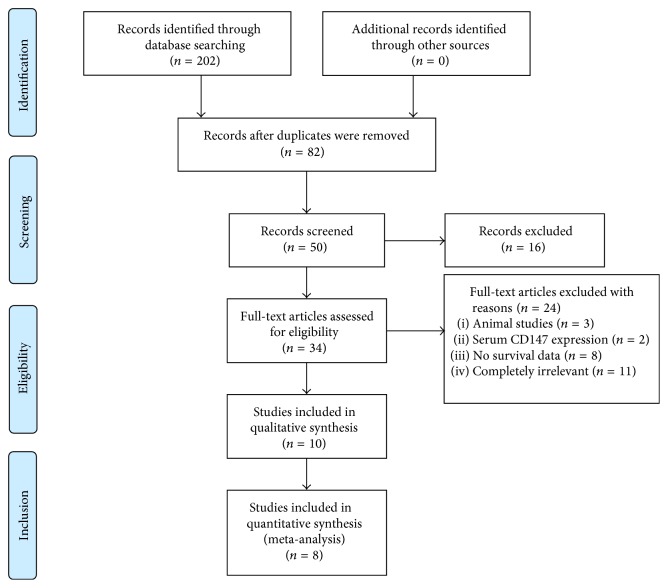
Flow chart of the selection process.

A total of 202 studies were identified, and 120 studies were excluded because of duplication.  Figure 1 illustrates the trial flow chart. After reading the titles and abstracts, 48 studies were excluded. Thirty-four full-text studies were carefully reviewed (excluded for being animal studies [*n* = 3], serum CD147 expression [*n* = 2], no survival data [*n* = 8], and being completely irrelevant [*n* = 11]). A total of 10 studies [18, 21, 26–31] were identified for qualitative analysis. The study by Ji et al. [32] did not provide the data of HR and 95% CI for HCC patients, which only know mean survival time of recurrence-free survival (RFS); another study (W-C Tsai) [33] does not provide the cut-off value for judging CD147 positive expression. After selection, 8 studies with 880 patients were finally used for analysis of the prognostic value of CD147 expression in the meta-analysis. All 8 studies adopted immunohistochemistry (IHC) as the detection method, but the method for judging negative and positive staining was different among them. In addition, all patients in eight studies were diagnosed with HCC (hepatocellular carcinoma) and were of Asian origin. Four studies reported OS (overall survival), four studies provided DFS/RFS (disease-free survival/recurrence-free survival), three studies reported median survival time, seven studies provided follow-up time, three articles contained HR from multivariate factors, and five articles provided survival curves. One of the two articles that talked about liver transplantation had the sample taken before the transplantation without radiotherapy and chemotherapy, whereas the other had studied specimens from liver cancer patients with cirrhosis. All samples from these two studies were confirmed for HCC by histological studies.  Table 1 lists the major characteristics of the selected studies; we used the NOS scale to evaluate the literature, and all of the studies had a score greater than 5, indicating that the quality of the literature was high. Moreover, we performed a subgroup analysis according to four aspects: follow-up time more than 5 years, HR from multivariate or univariate analysis, with or without liver transplantation, and cut-off value (more than 10% of cells stained). The characteristics of the studies are presented in Table 1 and the NOS results are presented in Table 2.

### 3.2. Correlation between CD147 Expression and OS

**Figure 2:**
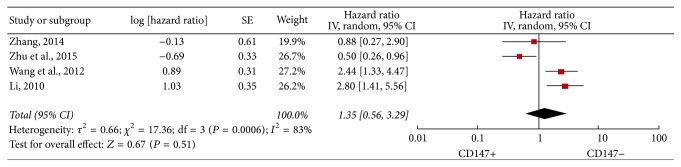
Forest plot of HR of OS for patients with HCC.

**Figure 3:**
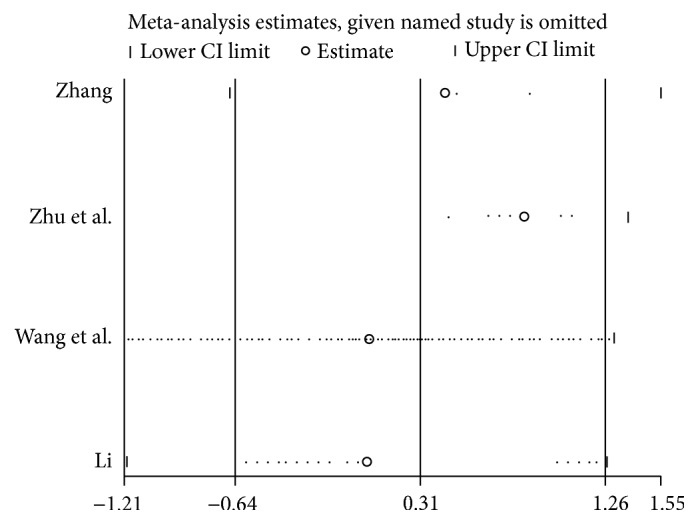
Sensitive analysis of OS for patients with HCC.

Four [18, 29, 30, 34] OS-related pieces of data displayed heterogeneity (*I*^2^ = 83%; *P* = 0.0006) and random model showed that high CD147 expression was not significantly associated with poor OS, as compared to low CD147 expression (HR = 1.35; 95% CI: 0.56–3.29; *P* = 0.51). In addition, we conducted subgroup analysis according to follow-up time, HR from multivariate or univariate analysis, with or without liver transplantation, and cut-off value (Table 3). In the univariate/multivariate subgroup analysis, heterogeneity was considerably dissolved in the univariate analysis group (*I*^2^ = 48%; *P* = 0.15). Moreover, there was a close association between OS and CD147 expression (HR = 2.21; 95% CI: 1.44–3.38; *P* = 0.0003) (Figure 2), but this result could not have enough persuasion due to the limitation of subgroup analysis. However, there were no significant differences in the subgroups of follow-up time more than 5 years (HR = 1.75; 95% CI: 0.57–5.34; *P* = 0.33) and without liver transplantation (HR = 1.35; 95% CI: 0.56–3.29; *P* = 0.51). In addition, sensitivity analysis indicated that the result was stable (Figure 3).

### 3.3. Correlation between CD147 Expression and DFS/RFS

**Figure 4:**
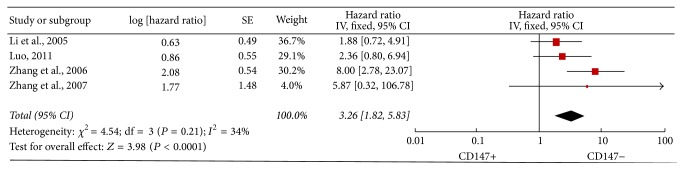
Forest plot of HR for DFS/RFS of patients with HCC.

**Figure 5:**
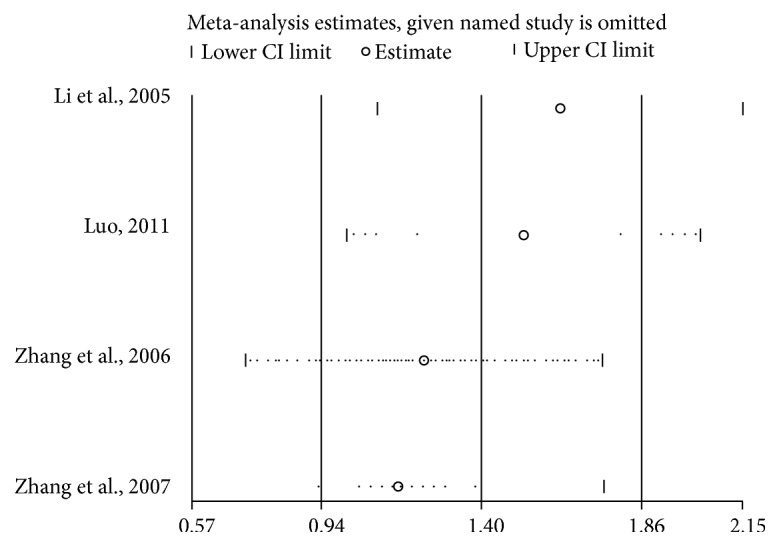
Sensitive analysis for DFS/RFS of patients with HCC.

Four studies [21, 27, 28, 31] demonstrated the association of CD147 expression with DFS/RFS. The combined data showed significant association between high CD147 expression and DFS/RFS (HR = 3.26; 95% CI: 1.82–5.83; *P* < 0.0001) without heterogeneity (*I*^2^ = 34%; *P* = 0.21) (Figure 4). Sensitivity analysis showed that our results were unstable (Figure 5). In addition, subgroup analysis indicated that there is significant difference in the groups of multivariate analysis, follow-up time less than 5 years, and with liver transplantation (Table 3). Thus, the association of CD147 expression with DFS/RFS of patients with HCC is speculative.

### 3.4. Correlation between CD147 Expression and Median Survival Time

Three studies [29, 30, 34] were chosen for analyzing the relationship between CD147 expression and median survival time in patients with HCC. There was significant association of high CD147 expression with median survival time (MSR = 0.336; 95% CI: 0.224–0.504; *P* = 0.000) with significant heterogeneity (*I*^2^= 92.1%; *P* = 0.000) (Appendix  2). Owing to the significant heterogeneity and the fact that only three studies were included, we also made a description of the results. Median survival times reported by Wang et al. [29], Li [30], and Zhang [34] were 24 months, 14 months, and 10 months, respectively, in high CD147 expression group. All these Zhang et al.'s studies have significant difference between the high CD147 expression and low CD147 expression groups with respect to median survival time. Therefore, the conclusion that high CD147 expression group has a shorter median survival time than low CD147 expression is speculative.

### 3.5. Correlation between CD147 Expression and Clinicopathological Parameters

**Figure 6:**
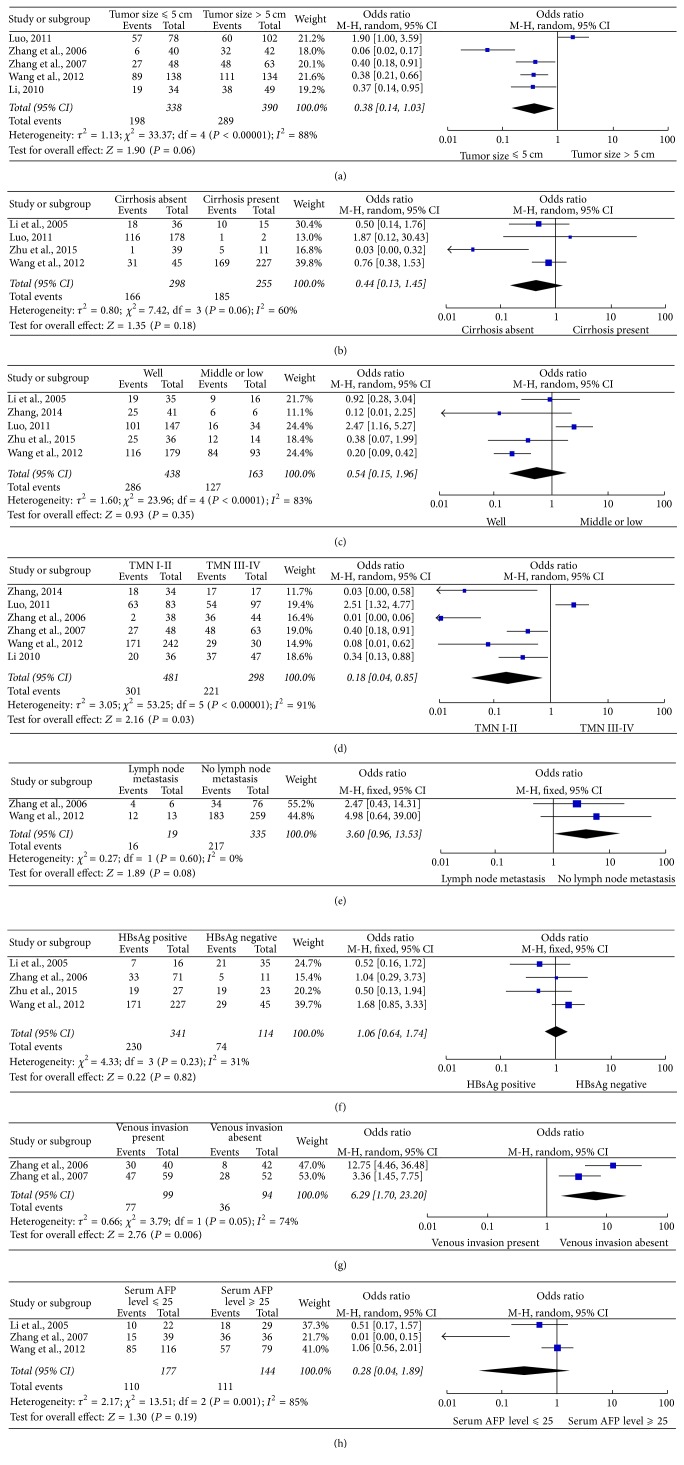
Association of CD147 expression with clinicopathological parameters.* Notes. *(a) The forest plot for the overall association between CD147 expression and tumor stage in patients with HCC. CD147 expression was not associated with tumor size of HCC cancer (OR = 0.38; 95% CI: 0.14–1.03; *P* = 0.06). (b) The forest plot for the overall association between CD147 expression and cirrhosis in patients with HCC. CD147 expression was not associated with cirrhosis of HCC cancer (OR = 0.4; 95% CI: 0.13–1.45; *P* = 0.18). (c) The forest plot for the overall association between CD147 expression and differentiation in patients with HCC. CD147 expression was not associated with differentiation of HCC cancer (OR = 0.54; 95% CI: 0.15–1.96; *P* = 0.35). (d) The forest plot for the overall association between CD147 expression and TMN in patients with HCC. CD147 expression was associated with the TNM stage of HCC cancer (OR = 0.18; 95% CI: 0.04–0.85; *P* = 0.03). (e) The forest plot for the overall association between CD147 expression and lymph node metastasis in patients with HCC. CD147 expression was not associated with lymph node metastasis of HCC cancer (OR = 3.60; 95% CI: 0.96–13.53; *P* = 0.06). (f) The forest plot for the overall association between CD147 expression and HBsAg in patients with HCC. CD147 expression was not associated with HBsAg of HCC cancer (OR = 1.06; 95% CI: 0.64–1.74; *P* = 0.82). (g) The forest plot for the overall association between CD147 expression and venous invasion in patients with HCC. CD147 expression was associated with venous invasion of HCC cancer (OR = 6.29; 95% CI: 1.70–23.20; *P* = 0.006). (h) The forest plot for the overall association between CD147 expression and serum AFP level in patients with HCC. CD147 expression was not associated with serum AFP level of HCC cancer (OR = 0.28; 95% CI: 0.04–1.89; *P* = 0.19).

Based on the ORs derived from each available study, we also evaluated the correlation between CD147 expression and some clinical characteristics, including tumor size, cirrhosis, differentiation, the TNM stage, lymph node metastasis, HBsAg, venous invasion, and serum AFP level. The results showed that CD147 expression was associated with the TNM stage (OR = 0.18; 95% CI: 0.04–0.85; *P* = 0.03) and venous invasion (OR = 6.29; 95% CI: 1.70–23.20; *P* = 0,006) (Table 4). However, there were no significant differences between CD147 expression and any other clinical characteristics (Figure 6).

## 4. Publication Bias

The publication bias of the included studies was evaluated through Egger's tests. The corresponding *P* values of OS and DFS/RFS were 0.782 (Appendix  3) and 0.608 (Appendix  4), respectively, indicating that the meta-analysis did not display publication bias.

## 5. Discussion

Hepatocellular carcinoma (HCC) is the most common primary liver cancer and the second most frequent cause of cancer-related death worldwide [35]. Caudron et al. reported that CD147 expression and ulceration status contributed to the overall survival of patients with cutaneous melanoma [36]. In addition, Bauman et al. demonstrated that membrane-associated CD147 expression was associated with tumor progression [37].

Our meta-analysis is the first one to investigate the association between CD147 expression and the survival rate of patients with liver cancer. A total of 880 patients with HCC were included in our meta-analysis. Our results indicated that there was no significant difference between CD147 expression and OS. However, the results differed among the subgroups of univariate analysis, which showed a close association between CD147 expression and OS. Tsai et al. [33] also demonstrated that CD147 expression was closely related to OS in univariate analysis. Moreover, low CD147 expression was related to longer survival. There exist conflicting views like those of Zhu et al. [18] who provided evidence that patients with HCC with high CD147 expression have longer survival. Our analyses also proved that CD147 expression in HCC is associated with DFS/RFS. This is contradictory to the report by Li [30], which showed that there was no significant difference in disease-free survival between high and low CD147 expression groups. According to our subgroup analysis, we found close association between CD147 expression and DFS/RFS in the multivariate analysis groups, groups with follow-up time less than 5 years, and with liver transplantation. However, further studies are warranted to extend the significance of these results. Our results indicate that patients with low CD147 expression have longer survival time than those with low CD147 expression with huge heterogeneity (*I*^2^ = 92.1%; *P* = 0.000). All three reports [29, 30, 34] indicated that high CD147 expression group had a shorter median survival time as compared to the low CD147 expression group.

OR for the TNM stage and venous invasion were statistically significant in the correlation study of CD147 expression with the clinical characteristics of patients. Although Zhang et al. [28] and Wang et al. [29] also reported that CD147 expression was closely related to the TNM stage, the results are speculative due to large heterogeneity (*I*^2^ = 91%; *P* < 0.00001). Moreover, Li [30] also demonstrated that the expression of CD147 was not associated with serum AFP level, tumor size, and differentiation.

In addition, Tsai et al. [33] demonstrated that the survival rate of the group with EMMPRIN score ≥ 200 was not significantly different from that of the group with EMMPRIN score < 200 (*P* = 0.35). Another study by Ji [32] found that there was no significant difference between high and low CD147 expression groups and mean survival time of RFS. In addition, Lee et al. [20] indicated that only the group with sCD147 levels > 24 ng/mL has a significant difference in 90-day survival and 180-day survival compared to sCD147 levels ≤ 24 ng/mL.

It should be noted that there are some limitations to the analyses presented here. Firstly, publication bias can be a concern because more positive results tend to get published, thus potentially exaggerating the association between CD147 expression and poor outcomes. Secondly, in the meta-analysis, HRs and 95% CI were directly extracted from original data from the three included studies. For other studies, HR had to be extrapolated from the survival curve, implying that the estimated HR may be less reliable than when directly obtained from published statistics. Thirdly, the studies have subjects of different age, follow-up time, and cut-off values. In addition, all patients in these included studies were of Asian origin. Moreover, the quality of some of the included studies was not completely satisfactory. These factors could also have affected the outcome of our evaluation of the prognostic value of CD147.

## 6. Conclusion

Despite the limitations of the present study and heterogeneity across the included studies, our systematic review and meta-analysis suggest that high CD147 expression may be related to the survival, TNM stage, and venous invasion in patients with HCC.

## Supplementary Material

Figure S1: Correlation between CD147 expression and median survival time. Appendix 1: Search strategies for this article.

## Figures and Tables

**Table 1:** Main characteristics of enrolled studies.

First author	Time	Country	Age	Number of patients	Survival results	Test method	Tumor types	HR	Follow-up time	Cancer stage	Expression location	Cut-off value	NOS score
*Li*	*2005*	China	24–69	51	DFS	IHC	HCC	Univariate	5–90 months	NA	Cell surface	Positive: more than 10% of cells stained	7
Zhang^*∗*^	2006	China	48.52 ± 9.60	82	RFS	IHC	HCC	Multivariate	1–45 months	TNMI-*χ*	Cell surface	Positive: more than 10% of cells stained	7
Zhang	2007	China	24–66	111	RFS	IHC	HCC	Multivariate	1–63 months	TNMI-*χ*	Cell surface	NA	7
Wang	2012	China	28–65	272	OS, MST	IHC	HCC	Univariate	NA	TNMI-*χ*	Cell surface	Positive: scores^#^ ≥ 2 were positive	7
Li	2010	China	15–65	83	OS, MST	IHC	HCC	Univariate	1–101 months	TNMI-*χ*	Cell surface	Positive: more than 10%	6
Luo^*∗*^	2011	China	NA	180	RFS	IHC	HCC	Univariate	0–12 months	TNMI-*χ*	Cell surface	Positive: more than 5%	6
Zhang	2014	China	24–66	51	OS, MST	IHC	HCC	Univariate	2–60 months	TNMI-*χ*	Cell surface	NA	6
Zhu	2015	China	31–76	50	OS	IHC	HCC	Multivariate	0–48 months	NA	Cell surface	Positive: more than 5%	7

MST: median survival time; *∗*: the research is liver transplantation; scores^#^: The positive cells were classified into 4 grades on the basis of the percentage of the positive cells: the number of the cells below 5% was zero score, 6%–25% was 1 score, 26%–50% was 2 score, and higher than 51% was 3 score; second the positive cells were classified into 4 grades on the basis of intensity of color: colorless was zero score, yellow was 1 score, light brown was 2 score, and deep brown was 3 score. Then the two kinds of scores were added: the score which was lower than 1 was negative and that which was higher than 2 was positive.

**Table 2:** NOS score of included studies.

Column	Entries	First author							
		Li, 2005	Zhang, 2006	Zhang, 2007	Wan, 2012	Li, 2010	Luo, 2011	Zhan, 2014	Zhu, 2015
Section	Is the definition adequate	☆	☆	☆	☆	☆	☆	☆	☆
	Representativeness of the cases	☆	☆	☆	☆	☆	☆	☆	☆
	Selection of controls	☆				☆	☆		
	Definition of controls	☆	☆	☆	☆	☆	☆	☆	☆

Comparability	Comparability of cases and controls on the basis of the design and analysis	☆	☆	☆	☆	☆	☆	☆	☆
	Ascertainment of exposure	☆	☆	☆	☆				☆

Exposure	Same method of ascertainment for cases and controls	☆	☆	☆	☆			☆	☆
	Nonresponse rate		☆	☆	☆	☆	☆	☆	☆

Total scores		7	7	7	7	6	6	6	7

**Table 3:** Results of subgroups analysis.

	OS						DFS/RFS					
	Number	HR (95% CI)	*P*_h_	*I* ^2^ (%)	*P*	Model	Number	HR (95% CI)	*P*_h_	*I* ^2^ (%)	*P*	Model
Total	4						4					
Univariate/Multivariate												
Multivariate	1	0.5 (0.26, 0.96)			0.04		2	7.72 (2,86, 20.86)	0.84	0	<0.0001	Fixed
Univariate	3	2.21 (1.44, 3.38)	0.15	48	0.0003	Fixed	2	2.08 (1.01, 4.26)	0.75	0	0.05	Fixed
Follow-up time												
More than 5 years	2	1.75 (0.57, 5.34)	0.1	63	0.33	Random	2	2.10 (0.84, 5.23)	0.46	0	0.11	Fixed
Less than 5 years	1	0.50 (0.26, 0.96)			0.04		2	4.40 (2,07, 9.36)	0.11	60	0.0001	Fixed
Liver transplantation												
Yes	0						2	4.40 (2.07, 9.36)	0.11	60	0.0001	Fixed
No	4	1.35 (0.56, 3.29)	0.0006	83	0.51	Random	2	2.10 (0.84, 5.23)	0.46	0	0.11	Fixed
Cut-off value												
More than 10% of cells stained	1	2.80 (1.41, 5.56)			0.03		2	3.81 (0.92, 15.76)	0.05	75	0.07	Random
Less than 10% of cells stained	1	0.50 (0.26, 0.96)			0.04		2	2.64 (0.96, 7.25)	0.56	0	0.06	Fixed

*P*_h_ means the heterogeneity of *P* value.

**Table 4:** Meta-analyses of CD147 Expression classified by clinicopathological parameters.

Variables	Number of studies	Model	OR (95% CI)	*P* value	Heterogeneity (*I*^2^, *P* value)
Tumor size (≤5 cm/>5 cm)	5	Random	0.38 (0.14, 1.03)	0.06	88%, <0.00001
Cirrhosis (absent/present)	4	Random	0.44 (0.13, 1.45)	0.18	60%, 0.06
Differentiation (well/middle or low)	5	Random	0.54 (0.15, 1.96)	0.35	83%, <0.0001
TMNI-*α*/*β*-*χ*	6	Random	0.18 (0.04, 0.85)	0.03	91%, <0.00001
Lymph node metastasis (yes/no)	2	Fixed	3.60 (0.96, 13.53)	0.06	0%, 0.60
HBsAg (positive/negative)	4	Fixed	1.06 (0.64, 1.74)	0.82	31%, 0.23
Venous invasion (present/absent)	2	Random	6.29 (1.70, 23.20)	0.006	74%, 0.05
Serum AFP level (≤25/≥25*μ*g/L)	3	Random	0.28 (0.04, 1.89)	0.19	85%, 0.001
