# Supplementary figures and images for: CD147 as a Novel Prognostic Biomarker for Hepatocellular Carcinoma: A Meta-Analysis
Source: Biomed Res Int. 2017 Mar 12;2017:5019367. doi: 10.1155/2017/5019367 (PMC5366185; doi:10.1155/2017/5019367)

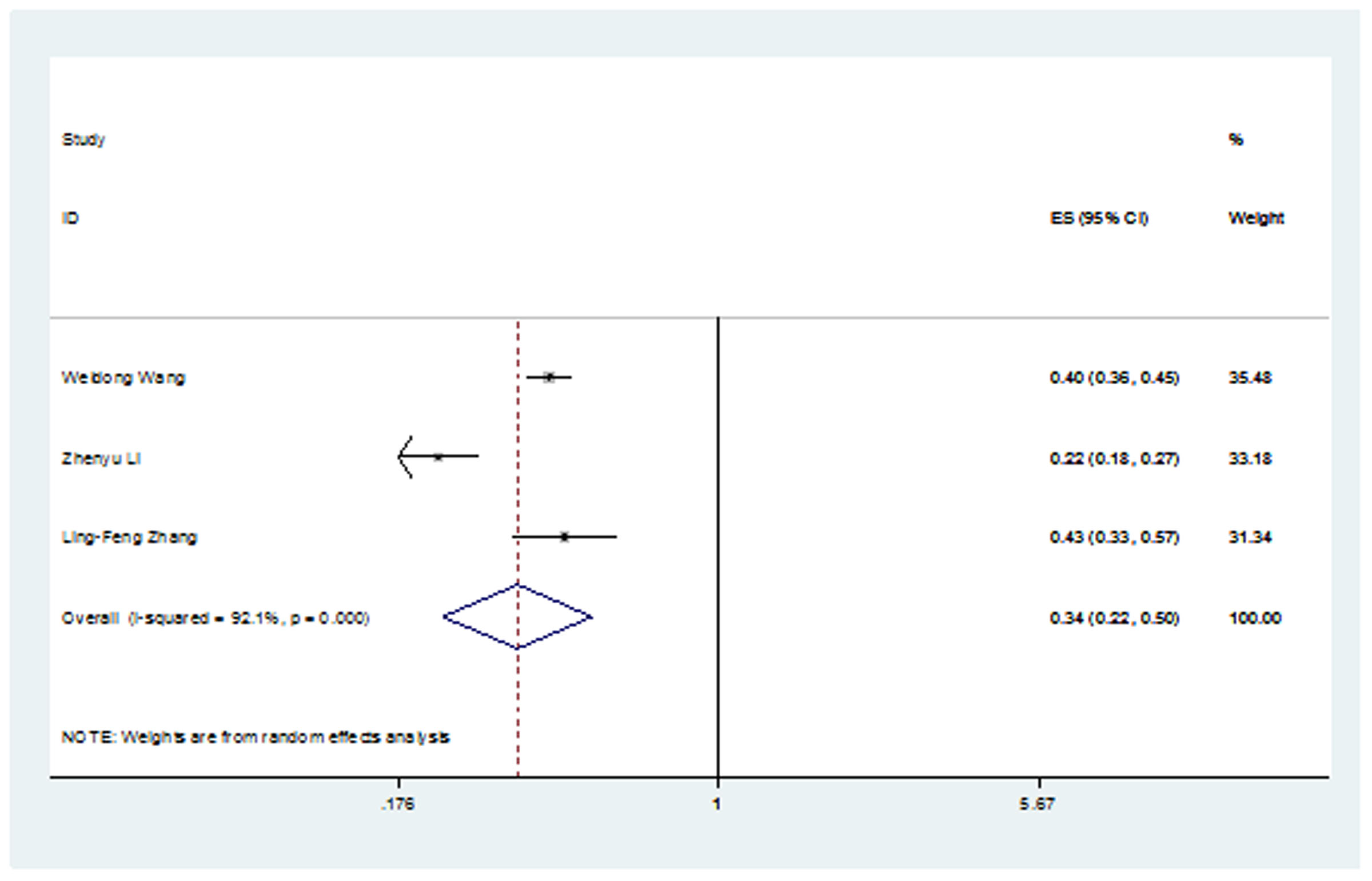

Supplement: Supplementary file 1 — Figure S1: Correlation between CD147 expression and median survival time. Appendix 1: Search strategies for this article. [file 5019367.f1.jpg]
