# Supplementary material for: CD147 as a Novel Prognostic Biomarker for Hepatocellular Carcinoma: A Meta-Analysis
Source: Biomed Res Int. 2017 Mar 12;2017:5019367. doi: 10.1155/2017/5019367 (PMC5366185; doi:10.1155/2017/5019367)
Supplement: Supplementary file 2 [file 5019367.f2.docx]

Appendix 1

1. Pubmed

24 articles

CD147

antigens, cd147[MeSH Terms] OR cd147[Title/Abstract] OR (extracellular[Title/Abstract] AND matrix[Title/Abstract] AND metalloproteinase[Title/Abstract] AND inducer[Title/Abstract]) OR "extracellular matrix metalloproteinase inducer"[Title/Abstract] OR EMMPRIN[Title/Abstract]

Liver cancer

Liver Neoplasms[MeSH Terms] OR (liver[Title/Abstract] AND neoplasm*[Title/Abstract]) OR (hepatic[Title/Abstract] AND neoplasm*[Title/Abstract]) OR (hepatocellular[Title/Abstract] AND cancer*[Title/Abstract]) OR (hepatic[Title/Abstract] AND cancer*[Title/Abstract]) OR (liver[Title/Abstract] AND cancer*[Title/Abstract])

prognosis

prognosis[MeSH Terms] OR survival[MeSH Terms] OR prognostic[Title/Abstract] OR prognosis[Title/Abstract] OR outcome[Title/Abstract] OR survival[Title/Abstract]

Total search method

(antigens, cd147[MeSH Terms] OR cd147[Title/Abstract] OR (extracellular[Title/Abstract] AND matrix[Title/Abstract] AND metalloproteinase[Title/Abstract] AND inducer[Title/Abstract]) OR "extracellular matrix metalloproteinase inducer"[Title/Abstract] OR EMMPRIN[Title/Abstract]) AND (Liver Neoplasms[MeSH Terms] OR (liver[Title/Abstract] AND neoplasm*[Title/Abstract]) OR (hepatic[Title/Abstract] AND neoplasm*[Title/Abstract]) OR (hepatocellular[Title/Abstract] AND cancer*[Title/Abstract]) OR (hepatic[Title/Abstract] AND cancer*[Title/Abstract]) OR (liver[Title/Abstract] AND cancer*[Title/Abstract])) AND (prognosis[MeSH Terms] OR survival[MeSH Terms] OR prognostic[Title/Abstract] OR prognosis[Title/Abstract] OR outcome[Title/Abstract] OR survival[Title/Abstract])

1. Embase

45 articles

('CD147 antigen'/exp OR cd147:ti,ab OR (extracellular:ti,ab AND matrix:ti,ab AND metalloproteinase:ti,ab AND inducer:ti,ab) OR "extracellular matrix metalloproteinase inducer":ti,ab OR EMMPRIN:ti,ab) AND ('liver tumor'/exp OR (liver:ti,ab AND neoplasm*:ti,ab) OR (hepatic:ti,ab AND neoplasm*:ti,ab) OR (hepatocellular:ti,ab AND cancer*:ti,ab) OR (hepatic:ti,ab AND cancer*:ti,ab) OR (liver:ti,ab AND cancer*:ti,ab)) AND ('prognosis'/exp OR 'survival'/exp OR prognostic:ti,ab OR prognosis:ti,ab OR outcome:ti,ab OR survival:ti,ab) AND [embase]/lim

1. Cochrane Library

4 articles

([mh "Antigens, CD147"] OR cd147:ti,ab OR (extracellular:ti,ab AND matrix:ti,ab AND metalloproteinase:ti,ab AND inducer:ti,ab) OR "extracellular matrix metalloproteinase inducer":ti,ab OR EMMPRIN:ti,ab) AND ([mh "Liver Neoplasms"] OR (liver:ti,ab AND neoplasm*:ti,ab) OR (hepatic:ti,ab AND neoplasm*:ti,ab) OR (hepatocellular:ti,ab AND cancer*:ti,ab) OR (hepatic:ti,ab AND cancer*:ti,ab) OR (liver:ti,ab AND cancer*:ti,ab)) AND ([mh prognosis] OR [mh survival] OR prognostic:ti,ab OR prognosis:ti,ab OR outcome:ti,ab OR survival:ti,ab)

1. WOS

59 articles

(TS=Antigens, CD147 OR TS=cd147 OR (TS=extracellular AND TS=matrix AND TS=metalloproteinase AND TS=inducer) OR TS="extracellular matrix metalloproteinase inducer" OR TS=EMMPRIN) AND ((TS=liver AND TS=neoplasm*) OR (TS=hepatic AND TS=neoplasm*) OR (TS=hepatocellular AND TS=cancer*) OR (TS=hepatic AND TS=cancer*) OR (TS=liver AND TS=cancer*)) AND (TS=prognostic OR TS=prognosis OR TS=outcome OR TS=survival)

5、CNKI

52 articles

CD147

SU='CD147' OR SU='antigen, CD147' OR SU='Extracellular matrix metalloproteinase inducer'

肝癌

SU='Liver neoplasms' OR SU='Liver neoplasm' OR SU='Liver cancer' OR SU='Hepatocellular carcinoma' OR SU='Liver metastasis'

预后

SU='prognosis' OR SU='survival'

总检索式

(SU='CD147' OR SU='antigen, CD147' OR SU='Extracellular matrix metalloproteinase inducer') AND (SU='Liver neoplasms' OR SU='Liver neoplasm' OR SU='Liver cancer' OR SU='Hepatocellular carcinoma' OR SU='Liver metastasis') AND ('prognosis' OR SU='survival')

6、WANFANG

58 articles

(subject:(CD147) + subject:(antigen, CD147) + subject:(Extracellular matrix metalloproteinase inducer)) * (subject:(Liver neoplasms) + subject:(Liver neoplasm) + subject:(Liver cancer) + subject:(Hepatocellular carcinoma) + subject:(Liver metastasis)) * (subject:(prognosis) + subject:(survival))

Appendix 2

Egger's test of OS

------------------------------------------------------------------------------

Std_Eff | Coef. Std. Err. t P>|t| [95% Conf. Interval]

-------------+----------------------------------------------------------------

slope | 1.011348 2.028188 0.50 0.667 -7.715239 9.737934

bias | -1.724275 5.453226 -0.32 0.782 -25.18761 21.73906

Appendix 3

Egger's test of DFS/RFS

Std_Eff | Coef. Std. Err. t P>|t| [95% Conf. Interval]

-------------+----------------------------------------------------------------

slope | 2.823975 2.389146 1.18 0.359 -7.45569 13.10364

bias | -3.060122 5.075003 -0.60 0.608 -24.8961 18.77585
